# Feline-Derived *Ligilactobacillus agilis* ZY25 and *Ligilactobacillus salivarius* ZY35 Alleviate *Enteropathogenic Escherichia coli*-Induced Intestinal Injury and Microbial Dysbiosis in Mice

**DOI:** 10.3390/microorganisms14030679

**Published:** 2026-03-17

**Authors:** Weiwei Wang, Li Pan, Chengyi Miao, Qianqian Chen, Huakai Wang, Chenxiang Sun, Xiaohan Chang, Yuqiang Zhang, Jianmei Wang, Wei Xiong

**Affiliations:** 1 Food Laboratory of Zhongyuan, Luohe 462300, China; bingzhi213608@163.com (W.W.); 18437959120@163.com (L.P.); miaochengyi@zyfoodlab.com (C.M.); 2020920269@stu.haut.edu.cn (Q.C.); huakaiwhk@163.com (H.W.); chenxiang_sun@163.com (C.S.); changxiaohan@zyfoodlab.com (X.C.); zhangyuqiangyu@163.com (Y.Z.); 2 Key Laboratory of Precision Nutrition and Food Quality, Department of Nutrition and Health, China Agricultural University, Beijing 100083, China; 15652509689@163.com

**Keywords:** feline-derived probiotics, *Enteropathogenic Escherichia coli*, intestinal barrier function, immune modulation, gut microbiota

## Abstract

*Enteropathogenic Escherichia coli* (EPEC) disrupts intestinal barrier integrity, induces inflammation, and alters gut microbial balance, leading to diarrhea and growth impairment. Probiotics are considered promising alternatives to antibiotics for managing enteric infections, yet the functional properties and underlying mechanisms of feline-derived strains remain unclear. This study evaluated the protective effects of *Ligilactobacillus* (*L.*) *agilis* ZY25 and *L. salivarius* ZY35, isolated from healthy cats, against EPEC-induced intestinal injury in C57BL/6 mice, with a focus on barrier function, immune modulation, and microbial homeostasis. In this 21-day experiment, 48 mice were assigned to six groups (*n* = 8/group): control, EPEC model (MOD), chlortetracycline treatment (CTC), probiotic treatment (PRO-T; post-infection only), probiotic pre-treatment (PRO-P; pre-infection only), and continuous probiotic supplementation (PRO; pre- and post-infection). EPEC challenge (0.2 mL; 1 × 10^9^ CFU/mL) was performed daily during experimental days 8–14. EPEC challenge resulted in weight loss (*p* < 0.05), increased (*p* < 0.05) diarrhea incidence, elevated (*p* < 0.05) serum D-lactate, diamine oxidase, and lipopolysaccharide levels, impaired intestinal morphology, immune imbalance, and microbial dysbiosis. Probiotic administration alleviated these alterations, as evidenced by restored intestinal morphology, reduced serum markers of barrier permeability (D-lactate, DAO, LPS), enhanced systemic immunoglobulins (IgA, IgG, IgM), a balanced cytokine profile (increased IL-4, IL-10; decreased TNF-α, IL-6, IL-1β, IFN-γ, CRP), and modulation of the gut microbiota (enrichment of beneficial taxa such as *Lachnospiraceae_NK4A136_group* and suppression of pro-inflammatory *Desulfovibrio*). The continuous supplementation regimen (PRO) produced the most consistent improvements among the three intervention strategies tested. These findings suggest that feline-derived probiotics mitigate EPEC-induced intestinal dysfunction, accompanied by improved barrier-related indices, immune rebalancing, and microbial stabilization, thereby providing proof-of-concept evidence for their further evaluation in feline gastrointestinal health.

## 1. Introduction

The intestinal tract is not only the primary site for digestion and nutrient absorption but also a critical immunological barrier that preserves homeostasis through coordinated interactions between the host and the microbiota [1]. A balanced gut microbiome supports epithelial integrity, immune regulation, and resistance to pathogen colonization, whereas dysbiosis is commonly linked to barrier breakdown, intestinal inflammation, and infectious diarrhea [2,3]. With growing concerns about antimicrobial resistance and antibiotic-associated disruption of commensal communities, probiotics have emerged as microbiome-preserving options for gastrointestinal management in humans and animals [4]. Probiotic bacteria can engage epithelial and immune cells to shape innate and adaptive responses and enhance host resistance to enteric infection [5,6].

*Enteropathogenic Escherichia coli* (EPEC) is a major cause of diarrheal disease across species. It adheres to intestinal epithelial cells via a type III secretion system and delivers effector proteins that disrupt the cytoskeleton and tight junctions, leading to increased permeability, inflammatory damage, and mucosal dysfunction [7,8]. These lesions are often accompanied by villus injury and increased circulating markers of barrier disruption, including diamine oxidase (DAO), D-lactate (D-LA), and lipopolysaccharide (LPS), reflecting epithelial compromise and endotoxin translocation [9]. Although antibiotics remain a conventional therapy, recurrent or prolonged use can further destabilize the microbiome and accelerate resistance, underscoring the need for adjunct or alternative strategies that preserve microbial homeostasis [10].

In this context, probiotic approaches are increasingly explored to counteract *E. coli* associated intestinal dysfunction. Selected lactobacilli can limit pathogen colonization and virulence while reinforcing epithelial barrier integrity. For instance, *L. plantarum* strains reduce *E. coli* adhesion and attenuate virulence-related responses, often accompanied by restoration of tight junction organization and improved barrier function in cellular and animal models [11,12]. *L. rhamnosus* GG also mitigates barrier-associated readouts and inflammatory signaling in *E. coli*-challenged mice [13]. Proposed mechanisms include competitive exclusion at mucosal niches, production of antimicrobial metabolites, stabilization of tight junction complexes, and immunomodulation through pattern recognition signaling, together with microbiota-derived metabolites that support epithelial repair [14,15,16]. However, most evidence is based on human- or rodent-associated strains, and host-adapted probiotic candidates for companion animals remain underexplored.

The feline gut microbiome exhibits marked host specificity and is shaped by obligate carnivory and distinctive metabolic features, which collectively influence microbial composition and functional potential and thereby affect gastrointestinal resilience and disease susceptibility [17]. This microbial ecosystem is highly sensitive to external perturbations, and stress, antimicrobial exposure, and abrupt dietary transitions frequently disrupt microbial homeostasis, increasing the risk of diarrhea and impairing mucosal barrier integrity [18]. Although the clinical evidence remains limited, controlled studies indicate that probiotic supplementation can reduce diarrhea incidence in high-risk settings and improve gut health-related outcomes, including metabolites derived from the microbiota and fecal inflammatory markers [19]. However, systematic mechanistic studies targeting feline-derived lactobacilli are still lacking, particularly with respect to their role in attenuating barrier dysfunction induced by EPEC [20].

Building on the previous isolation of two feline-derived strains, *L. agilis* ZY25 and *L. salivarius* ZY35, from healthy cat feces, which showed strong in vitro inhibition of EPEC largely mediated by lactic and acetic acid production [21], the present study sought in vivo evidence for their capacity to attenuate EPEC-induced intestinal injury. The primary objectives were: (1) to determine whether the timing and duration of probiotic administration (preventive, therapeutic, or continuous) influence efficacy against EPEC-induced intestinal injury; and (2) to assess barrier-related injury, systemic immune responses, intestinal histopathology, and cecal microbiota alterations in a murine EPEC challenge model. Using a murine EPEC challenge model with chlortetracycline as a positive control, we sought to establish a controlled first-step in vivo proof-of-concept system to evaluate the biological efficacy of these feline-derived strains and to compare different intervention regimens under standardized experimental conditions. Although this model does not replicate the full physiological and microbial context of the feline intestine, it provides a tractable mammalian platform for assessing barrier-related injury, immune responses, and microbiota alterations before target-species validation. These data are therefore intended to support further development and subsequent evaluation of feline-oriented probiotic formulations, rather than to serve as definitive evidence of efficacy in cats.

## 2. Materials and Methods

### 2.1. Animal Ethics Statement

All animal procedures were approved by the Institutional Animal Care and Use Committee (IACUC) of China Agricultural University (Approval No. Aw20704202-5-4) and were conducted in accordance with the guidelines of the Institute of Nutrition and Health, China Agricultural University.

### 2.2. Animals and Experimental Design

Six-week-old male C57BL/6 mice (20–25 g) were purchased from Beijing HFK Bioscience Co., Ltd. (Beijing, China). All animals were specific-pathogen-free (SPF) grade. Although baseline E. coli levels were not specifically quantified, using SPF-grade mice from a single vendor and a 7-day acclimatization period aimed to minimize inter-individual microbial variability prior to the experiment. The EPEC strain was kindly provided by China Agricultural University (Beijing, China). The probiotic preparation consisted of two feline-derived strains, *L. agilis* ZY25 and *L. salivarius* ZY35, isolated from feces of healthy cats. These strains had been previously characterized in our published study [21] for anti-EPEC activity as well as key safety- and probiotic-related properties, including acid tolerance, bile tolerance, antimicrobial susceptibility/safety assessment, and adhesion-related characteristics. Based on those prior evaluations, they were selected for the present in vivo study. For animal administration, the two strains were mixed at a 1:1 ratio and suspended in phosphate-buffered saline (PBS) to obtain a final concentration of 2 × 10^9^ colony-forming units (CFU)/mL. Chlortetracycline (CTC) was purchased from Zhongding Animal Pharmaceutical Co., Ltd. (Chizhou, China) and prepared in PBS at 1 mg/mL. Unless otherwise specified, all gavage solutions were prepared in PBS. Mice were used as a controlled mammalian model for initial in vivo evaluation because the EPEC challenge system is well established and allows standardized infection conditions, reduced inter-individual variability, and systematic assessment of intestinal barrier injury, inflammatory responses, and microbial changes prior to validation in the target species.

The study lasted 28 days, including a 7-day acclimatization period followed by a 21-day experimental period (experimental days 1–21; see Table 1 for the intervention schedule). After acclimatization, mice were randomly assigned to six groups (*n* = 8 per group) using a computer-generated random number table (Random Allocation Software, version 1.0). All animals completed the study, and no mice were excluded because of death, disease, or treatment-related complications during the experiment. Body weight and fecal score were monitored in all animals throughout the study. For terminal analyses, randomly selected subsets were used for some downstream assays, as detailed below. normal control (CON), EPEC-infected model (MOD), antibiotic positive control (CTC), probiotic treatment (PRO-T), probiotic pre-treatment (PRO-P), and probiotic prevention plus treatment (PRO). To minimize bias, all experimental procedures including gavage administration, sample collection, and subsequent outcome assessments (body weight, diarrhea scoring, histological analyses, serum assays, and 16S rRNA sequencing) were performed by investigators blinded to the group allocation. The blinding was maintained until after all data were collected and preliminary statistical analyses were completed. Cage locations were randomized within the animal facility to account for potential environmental gradients. During the experimental period, all mice were housed in a controlled environment with a 12 h light/dark cycle, ambient temperature of 22 ± 2 °C, and relative humidity of 50 ± 10%. Mice were provided ad libitum access to a standard laboratory chow diet and sterilized water throughout the study.

Mice were gavaged with 0.2 mL per administration according to a three-phase schedule, which is summarized in Table 1. Phase I (days 1–7) was the prevention/pre-feeding phase, during which probiotic pre-exposure was established in the relevant groups. Phase II (days 8–14) was the infection/challenge phase, during which mice in the challenged groups received EPEC daily. Phase III (days 15–21) was the treatment/recovery phase, during which post-challenge interventions were continued according to group assignment. The probiotic suspension was freshly prepared each day by mixing the two strains at a 1:1 ratio in sterile PBS to achieve a final concentration of 2 × 10^9^ CFU/mL. Gavage was performed using a sterile 20-gauge curved feeding needle between 9:00 and 11:00 AM each day to minimize circadian variations. For groups receiving both probiotic and EPEC on the same day (PRO-P and PRO during the challenge phase), probiotics were administered 2 h prior to EPEC gavage to allow for potential competitive exclusion effects.

Prevention/pre-feeding phase (experimental days 1–7): CON, MOD, CTC, and PRO-T received PBS once daily, whereas PRO-P and PRO received the probiotic mixture once daily (0.2 mL; 2 × 10^9^ CFU/mL). EPEC challenge phase (experimental days 8–14): CON continued to receive PBS, while MOD, CTC, PRO-T, PRO-P, and PRO were challenged daily with EPEC suspension (0.2 mL; 1 × 10^9^ CFU/mL). During this phase, PRO-P and PRO additionally received the probiotic mixture (0.2 mL; 2 × 10^9^ CFU/mL) 2 h prior to EPEC gavage each day. Treatment phase (experimental days 15–21): the CTC group received CTC once daily (0.2 mL; 1 mg/mL), PRO-T and PRO received the probiotic mixture once daily (0.2 mL; 2 × 10^9^ CFU/mL), and CON, MOD, and PRO-P received PBS once daily. Throughout all phases, the probiotic mixture was administered at a consistent volume of 0.2 mL, containing a total of 4 × 10^8^ CFU (2 × 10^8^ CFU of each strain) per dose. The concentration and dosage were based on preliminary experiments and previous studies demonstrating efficacy [21]. All mice were maintained under identical controlled environmental conditions with ad libitum access to diet and water throughout the study.

### 2.3. Sample Collection

At the end of the study (day 28), mice were anesthetized with isoflurane inhalation, and blood was collected via retro-orbital bleeding for serum preparation. The blood was transferred into sterile, dry tubes, allowed to clot at room temperature (approximately 20–25 °C) for 60 min, and then centrifuged at 3000 rpm for 15 min at 4 °C; the resulting serum was collected, aliquoted, and stored at −20 °C for subsequent biochemical and immunological analyses. Immediately thereafter, the abdominal cavity was opened, and the liver and spleen were excised, gently blotted dry, and weighed. The cecum was isolated, and cecal contents were collected into sterile tubes, kept on ice during handling, and stored at −80 °C for 16S rRNA gene sequencing and microbiota profiling. In parallel, segments of the jejunum and ileum were harvested, gently rinsed with cold PBS to remove residual luminal contents, and fixed in 4% paraformaldehyde for subsequent histological processing, including embedding, sectioning, and staining. Following completion of sample collection, animals were euthanized by cervical dislocation under deep anesthesia.

### 2.4. Body Weight Monitoring and Diarrhea Scoring

Body weight was recorded every other day using an electronic balance (Deante, JY5002; Tianjin, China). Diarrhea severity was evaluated at the same frequency using a fecal scoring system adapted from Wang et al. [22]: 0, normal stool; 1, altered stool color and/or consistency; 2, wet tail and/or mucus; and 3, liquid stools. A score ≥ 1 was considered indicative of diarrhea. At study termination, the liver and spleen were weighed using an analytical balance (Deante, FA124X; Tianjin, China). Organ indices were calculated as organ weight (g)/body weight (g) × 100%.

### 2.5. Histology and Morphometry

Histological analysis of the jejunum, ileum, and spleen was performed using hematoxylin and eosin (H&E) staining with reference to previously reported procedures [23]. Briefly, tissues were fixed in 4% paraformaldehyde, routinely processed for paraffin embedding, and sectioned (4–5 μm). Paraffin sections were deparaffinized and rehydrated, followed by H&E staining. Whole-slide images were acquired using a Pannoramic MIDI scanner (3DHISTECH, Budapest, Hungary). For the jejunum and ileum, villus height and crypt depth were measured on well-oriented villus–crypt units from H&E-stained sections using ImageJ software (version 1.52a; National Institutes of Health, Bethesda, MD, USA). Multiple villus–crypt units were assessed per section, and the mean value per mouse was used for statistical analysis. Spleen sections were evaluated histopathologically based on H&E-stained morphology, without morphometric measurements.

### 2.6. Serum Biochemical and Immunological Analyses

Serum inflammatory cytokines—including tumor necrosis factor-α (TNF-α), interleukin-1β (IL-1β), interleukin-4 (IL-4), interleukin-6 (IL-6), interleukin-10 (IL-10), and interferon-γ (IFN-γ)—as well as lipopolysaccharide (LPS), C-reactive protein (CRP), immunoglobulins (IgA, IgG, and IgM), and intestinal barrier-related markers (D-lactate and diamine oxidase) were determined in serum collected at study termination. Cytokines, D-lactate, and diamine oxidase were quantified using enzyme-linked immunosorbent assay (ELISA) kits, whereas immunoglobulins were measured by an immunoturbidimetric method; LPS and CRP were measured using the corresponding commercial assay kits. All assays were performed according to the manufacturers’ instructions using kits from Shanghai Enzyme Link Bio-technology Co., Ltd. (Shanghai, China).

### 2.7. 16S rRNA Gene Sequencing and Microbial Bioinformatics Analysis

Genomic DNA was extracted from cecal content samples collected from five randomly selected mice per group (*n* = 5 per group). Sample selection for 16S rRNA gene sequencing was performed without reference to clinical, biochemical, histological, or microbiota outcome data. DNA extraction was conducted using the MagPure Soil DNA LQ Kit (Shanghai Magen Biotechnology Co., Ltd., Shanghai, China) according to the manufacturer’s instructions. DNA quality and quantity were assessed by agarose gel electrophoresis and a NanoDrop spectrophotometer. The V3–V4 hypervariable regions of the bacterial 16S rRNA gene were amplified using primers 338F (5′-ACTCCTACGGGAGGCAGCAG-3′) and 806R (5′-GGACTACHVGGGTWT CTAAT-3′) on an ABI GeneAmp^®^ 9700 thermocycler (Applied Biosystems, Foster City, CA, USA). PCR products were checked by 2% agarose gel electrophoresis, and the target bands were excised and purified using the AxyPrep DNA Gel Extraction Kit (Axygen Biosciences, Union City, CA, USA) following the manufacturer’s protocol. Purified amplicons were quantified with a Quantus™ Fluorometer (Promega Corporation, Madison, WI, USA). For samples requiring replicate amplification, amplicons from the same sample were pooled in equimolar amounts prior to library preparation. Sequencing libraries were constructed using the NEXTflex™ Rapid DNA-Seq Kit (Bio Scientific, San Diego, CA, USA), and paired-end sequencing (2 × 300 bp) was performed on an Illumina MiSeq PE300 platform (Illumina, San Diego, CA, USA) following the standard protocols established by Majorbio Bio-Pharm Technology Co., Ltd. (Shanghai, China).

Raw FASTQ reads were demultiplexed and quality-filtered using fastp (v0.19.6) and merged using FLASH (v1.2.7) with standard criteria. Optimized sequences were clustered into operational taxonomic units (OTUs) at 97% sequence similarity using UPARSE (v11.0.667), and representative sequences were selected for downstream analyses. Taxonomic assignment of OTU representative sequences was performed using the Ribosomal Database Project (RDP) Classifier (v11.5) against the SILVA database (v138.2) with a confidence threshold of 0.7. Bioinformatic analyses were conducted using the Majorbio Cloud platform. Because the sequencing dataset in this study was originally processed using an established UPARSE-based 97% OTU workflow, all downstream community analyses were performed at the OTU level. Although ASV-based methods generally provide higher taxonomic resolution, the OTU-based approach used here was considered appropriate for overall community-level comparison in this proof-of-concept study. For LEfSe (Linear Discriminant Analysis Effect Size) analysis, the threshold on the logarithmic LDA score was set to 2.0 to identify significant discriminatory taxa among groups.

### 2.8. Statistical Analyses

The initial group size was *n* = 8 per group, and all animals completed the study without death or clinical exclusion. Body weight and fecal score were analyzed in all animals (*n* = 8/group). Because this study was designed as an initial proof-of-concept experiment and terminal biochemical/histological assays were constrained by assay resources, serum, organ index, and histological analyses were performed in a randomly selected subset of six mice per group (*n* = 6/group). Cecal microbiota analysis by 16S rRNA sequencing was performed in five randomly selected mice per group (*n* = 5/group) because of sequencing-resource limitations. Sample selection for downstream analyses was performed without reference to outcome measurements. The exact *n* for each analysis is indicated in the corresponding figure legends. All statistical analyses were performed using IBM SPSS Statistics (version 24.0; IBM Corp., Armonk, NY, USA). Data are presented as mean ± standard error of the mean (SEM) unless otherwise stated. Prior to parametric testing, normality and homogeneity of variance were evaluated. For endpoint measurements (e.g., serum indices, histological morphometry, and organ indices), comparisons among multiple groups were conducted using one-way analysis of variance (ANOVA) followed by Tukey’s honestly significant difference (HSD) test for post hoc multiple comparisons. For one-way ANOVA models, effect size was estimated as η^2^. In figures and tables, different lowercase letters indicate significant differences among groups (*p* < 0.05), whereas groups sharing at least one letter are not significantly different. For the fecal scoring index (ordinal data), differences between each treatment group and the MOD group at individual time points were analyzed using the non-parametric Mann–Whitney U test. Body weight and fecal scoring index plots were generated using OriginPro 2024 (OriginLab Corp., Northampton, MA, USA), and all other graphs were generated using GraphPad Prism (version 10.1.2; GraphPad Software, San Diego, CA, USA). For 16S rRNA gene sequencing data, downstream statistical analyses and visualizations were performed based on the OTU abundance table, including alpha-diversity and beta-diversity analyses; group differences in community structure were evaluated using permutational multivariate analysis of variance (PERMANOVA), and differential taxa were identified using Kruskal–Wallis tests or LEfSe. Where applicable, *p* values were adjusted for multiple testing using the Benjamini–Hochberg false discovery rate (FDR) procedure. A formal a priori power analysis was not performed; sample size was determined based on previous comparable animal studies, practical feasibility, and ethical considerations.

## 3. Results

### 3.1. Probiotics Mitigate Diarrhea and Weight Loss

**Figure 1:**
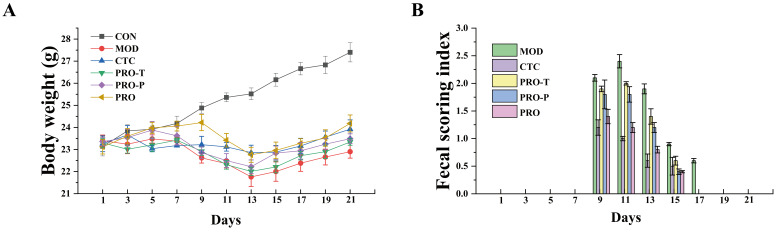
Effects of probiotic intervention on body weight and fecal score in EPEC-challenged mice. (**A**) Body weight changes (g) during the experimental period, (**B**) Time course of the fecal scoring index reflecting diarrhea severity (scoring criteria are described in the Methods). Data are presented as mean ± SEM (*n* = 8). CON, control; MOD, model; CTC, chlortetracycline; PRO-T, probiotic treatment; PRO-P, probiotic pre-treatment; PRO, probiotic prevention plus treatment; EPEC, *enteropathogenic Escherichia coli*.

During the pre-feeding phase (experimental days 1–7), body weights were comparable across groups and remained stable at approximately 23–24 g (Figure 1A). Following EPEC challenge (days 8–14), the CON group continued to gain weight, whereas all EPEC-exposed groups exhibited an attenuated weight-gain trajectory. By day 15, body weight reached 26.12 g in CON but decreased to 22.00 g in MOD, a 15.8% reduction (*p* < 0.05); the CTC, PRO-T, PRO-P, and PRO groups were 22.88, 22.21, 22.84, and 22.96 g, respectively, corresponding to reductions of 12.4%, 15.0%, 12.6%, and 12.1% versus CON (all *p* < 0.05; no significant differences among intervention groups at this time point) (Figure 1A). During the treatment/recovery phase (days 15–21), body weight increased in all groups. By day 21, CON remained the highest at 27.40 g and MOD remained the lowest at 22.90 g, while CTC, PRO-T, PRO-P, and PRO reached 23.92, 23.34, 23.49, and 24.18 g, respectively.

Diarrhea severity is summarized in Figure 1B. CON maintained a fecal score of 0 throughout the study. In contrast, all EPEC-challenged groups exhibited diarrhea episodes (score ≥ 1), mainly between days 9 and 15. MOD consistently showed the highest mean fecal scores during this interval, whereas PRO displayed lower mean scores than MOD and also tended to be lower than PRO-T and PRO-P. By day 17, diarrhea was only observed in MOD, while fecal scores in the other groups returned to 0. The detailed raw values and statistical comparisons corresponding to Figure 1A,B are provided in Supplementary Tables S1 and S2.

### 3.2. Probiotics Improve Serum Biomarkers Associated with Barrier Disruption

**Figure 2:**
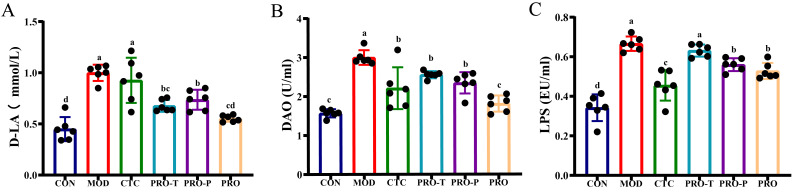
Probiotic intervention modulates serum biomarkers of intestinal permeability and endotoxemia in EPEC-challenged mice. (**A**) Serum D-lactate (D-LA, mmol/L), (**B**) Serum diamine oxidase (DAO, U/mL), (**C**) Serum lipopolysaccharide (LPS, EU/mL). Data are presented as mean ± SEM (*n* = 6). CON, control; MOD, model; CTC, chlortetracycline; PRO-T, probiotic treatment; PRO-P, probiotic pre-treatment; PRO, probiotic prevention plus treatment. Different lowercase letters above bars indicate significant differences among groups (*p* < 0.05).

Serum D-LA, DAO, and LPS were measured to evaluate EPEC-associated barrier disruption and endotoxin translocation (Figure 2A–C). Compared with CON, all three biomarkers were significantly increased in MOD (D-LA: 1.00 vs. 0.45 mmol/L; DAO: 3.00 vs. 1.57 U/mL; LPS: 0.67 vs. 0.34 EU/mL).

All interventions reduced these elevations to varying extents. For D-LA, levels decreased to 0.93, 0.68, 0.74, and 0.55 mmol/L in CTC, PRO-T, PRO-P, and PRO, corresponding to reductions of 7.0%, 32.0%, 26.0%, and 45.0% relative to MOD, respectively (Figure 2A). For DAO, levels decreased to 2.20, 2.55, 2.34, and 1.81 U/mL, representing reductions of 26.7%, 15.0%, 22.0%, and 39.7% versus MOD (Figure 2B). For LPS, levels decreased to 0.46, 0.63, 0.56, and 0.53 EU/mL in CTC, PRO-T, PRO-P, and PRO, corresponding to reductions of 31.3%, 6.0%, 16.4%, and 20.9% relative to MOD (Figure 2C).

### 3.3. Serum Immunoglobulins

**Figure 3:**
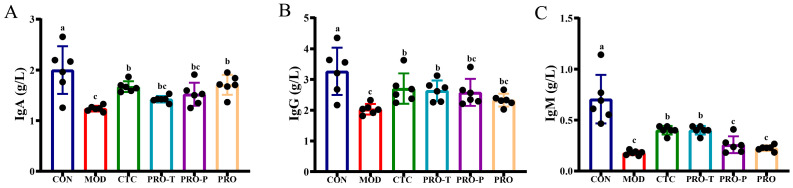
Effects of probiotic regimens on serum immunoglobulins in challenged mice. (**A**) Serum IgA (g/L), (**B**) Serum IgG (g/L), (**C**) Serum IgM (g/L). Data are presented as mean ± SEM (*n* = 6). CON, control; MOD, model; CTC, chlortetracycline; PRO-T, probiotic treatment; PRO-P, probiotic pre-treatment; PRO, probiotic prevention plus treatment. Different lowercase letters above bars indicate significant differences among groups (*p* < 0.05).

To profile humoral immune status, serum IgA, IgG, and IgM were determined in g/L (Figure 3A–C). Relative to CON, MOD exhibited a significant reduction in all three immunoglobulins. In MOD, IgA, IgG, and IgM were 1.24, 2.03, and 0.18 g/L, respectively, whereas the corresponding concentrations in CON were 2.00, 3.27, and 0.71 g/L. For IgA, concentrations increased to 1.67 g/L in CTC and 1.71 g/L in PRO, representing increases of 34.7% and 37.9% relative to MOD; PRO-T and PRO-P showed intermediate recovery to 1.42 and 1.52 g/L, corresponding to increases of 14.5% and 22.6% relative to MOD (Figure 3A). For IgG, levels increased to 2.71 g/L in CTC and 2.64 g/L in PRO-T, corresponding to increases of 33.5% and 30.0% relative to MOD; PRO-P and PRO also elevated IgG to 2.58 and 2.33 g/L, yielding increases of 27.1% and 14.8% relative to MOD (Figure 3B). For IgM, the most pronounced rebound was observed in CTC and PRO-T, both reaching 0.40 g/L, which is 122.2% higher than MOD; more modest increases were seen in PRO-P and PRO, with IgM at 0.26 and 0.23 g/L, corresponding to 44.4% and 27.8% higher than MOD (Figure 3C).

### 3.4. Serum Cytokines and CRP

**Figure 4:**
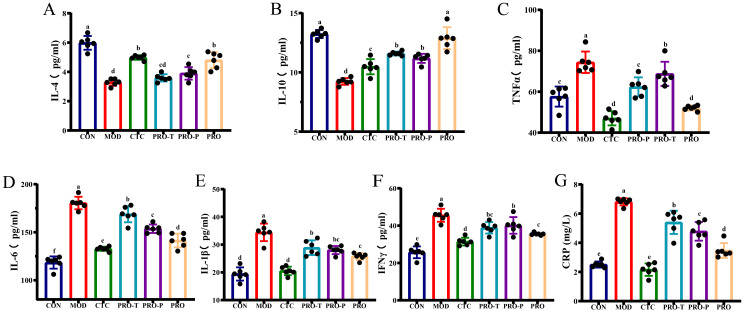
Serum cytokine profiles and CRP levels across experimental groups. (**A**) IL-4 (pg/mL), (**B**) IL-10 (pg/mL), (**C**) TNF-α (pg/mL), (**D**) IL-6 (pg/mL), (**E**) IL-1β (pg/mL), (**F**) IFN-γ (pg/mL), (**G**) CRP (mg/L). Data are presented as mean ± SEM (*n* = 6). CON, control; MOD, model; CTC, chlortetracycline; PRO-T, probiotic treatment; PRO-P, probiotic pre-treatment; PRO, probiotic prevention plus treatment. Different lowercase letters above bars indicate significant differences among groups (*p* < 0.05).

Serum cytokines IL-4, IL-10, TNF-α, IL-6, IL-1β, IFN-γ (all in pg/mL) and CRP (mg/L) were quantified to characterize systemic inflammatory status (Figure 4A–G). Compared with CON, MOD showed a significant decrease in the anti-inflammatory cytokines IL-4 and IL-10, with IL-4 at 3.28 pg/mL and IL-10 at 9.24 pg/mL in MOD versus 5.98 pg/mL and 13.24 pg/mL in CON, respectively. In parallel, MOD exhibited significantly higher pro-inflammatory readouts, including TNF-α at 74.40 pg/mL, IL-6 at 180.44 pg/mL, IL-1β at 34.43 pg/mL, IFN-γ at 45.57 pg/mL, and CRP at 6.81 mg/L, compared with 48.44 pg/mL, 118.50 pg/mL, 19.42 pg/mL, 25.74 pg/mL, and 2.51 mg/L in CON (*p* < 0.05).

Interventions shifted these markers away from the MOD profile to varying degrees. IL-4 increased to 4.80 pg/mL in PRO and 4.98 pg/mL in CTC (46.3% and 51.8% higher than MOD), and IL-10 increased to 12.89 pg/mL in PRO (39.5% higher than MOD), with smaller increases in PRO-T (11.59 pg/mL) and PRO-P (11.18 pg/mL). In parallel, TNF-α decreased to 52.02 pg/mL in PRO and 47.00 pg/mL in CTC (30.1% and 36.8% lower than MOD), with intermediate reductions in PRO-T and PRO-P. IL-6, IL-1β, and IFN-γ followed the same direction, decreasing by 21.6%, 25.2%, and 22.0% in PRO and by 26.4%, 40.4%, and 31.0% in CTC relative to MOD. Consistently, CRP declined to 3.47 mg/L in PRO and 2.16 mg/L in CTC (49.0% and 68.3% lower than MOD) (Figure 4G).

### 3.5. Spleen Morphology and Organ Indices

**Figure 5:**
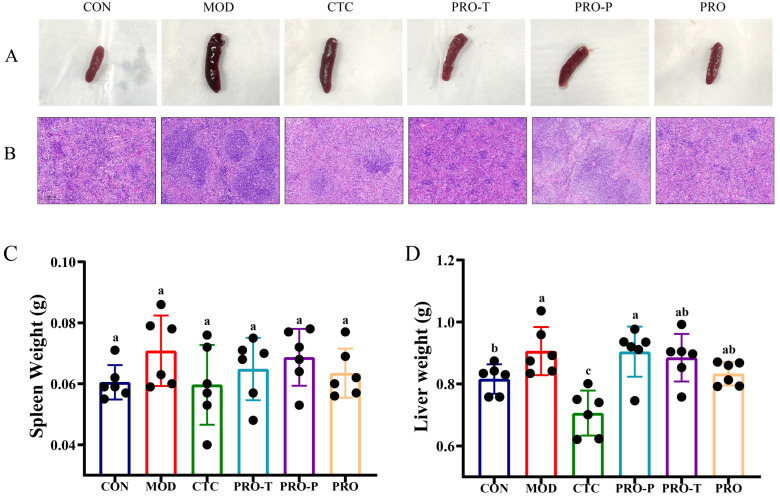
Effects of probiotic regimens on spleen morphology, splenic histology, and organ weights. (**A**) Representative gross appearance of spleen, (**B**) Representative H&E-stained spleen sections (scale bar = 0.200 mm), (**C**) Spleen weight (g), (**D**) Liver weight (g). Data are presented as mean ± SEM (*n* = 6). CON, control; MOD, model; CTC, chlortetracycline; PRO-T, probiotic treatment; PRO-P, probiotic pre-treatment; PRO, probiotic prevention plus treatment. Different lowercase letters above bars indicate significant differences among groups (*p* < 0.05).

Representative spleen photographs are shown in Figure 5A. Compared with CON, mice in MOD displayed an apparent increase in spleen size, whereas CTC and probiotic-treated groups showed a gross appearance closer to CON. Histological evaluation of spleen sections by H&E staining is presented in Figure 5B. In MOD, the splenic architecture appeared more disturbed, with less clearly demarcated white pulp regions and a denser cellular background suggestive of heightened immune activation. In contrast, CTC and probiotic regimens showed an overall improvement in tissue organization, with better-preserved white pulp structure and more uniform parenchymal appearance relative to MOD.

Quantitative organ indices are summarized in Figure 5C–D. Spleen weight was higher in MOD than in CON (0.071 and 0.061 g, respectively), corresponding to a 16.4% increase. In the intervention groups, spleen weight was 0.060 g in CTC, 0.065 g in PRO-T, 0.069 g in PRO-P, and 0.064 g in PRO, which represent decreases of 15.5%, 8.5%, 2.8%, and 9.9% relative to MOD, respectively (Figure 5C). Liver weight was also elevated in MOD compared with CON (0.91 and 0.82 g, respectively), corresponding to an 11.0% increase. CTC yielded the lowest liver weight at 0.71 g, reflecting a 22.0% decrease relative to MOD. Liver weight in PRO was 0.83 g, a reduction of 8.8% relative to MOD and close to the CON level, whereas PRO-T and PRO-P were 0.90 and 0.88 g, corresponding to decreases of 1.1% and 3.3% relative to MOD, respectively (Figure 5D).

### 3.6. Jejunal and Ileal Histomorphology

**Figure 6:**
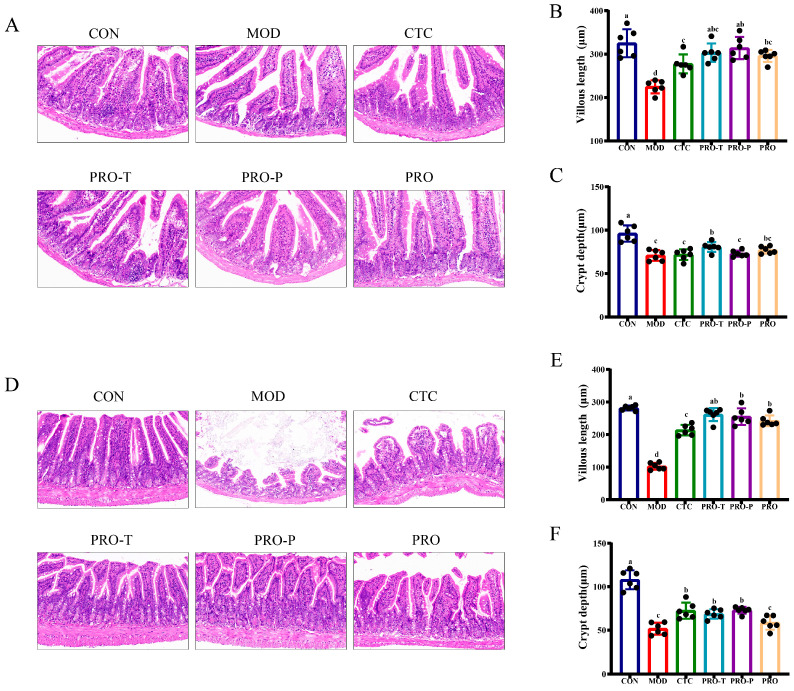
Probiotic regimens improve jejunal and ileal histomorphology following challenge. (**A**) Representative H&E-stained jejunal sections (scale bar = 0.100 mm), (**B**) Jejunal villus length (μm), (**C**) Jejunal crypt depth (μm), (**D**) Representative H&E-stained ileal sections (scale bar = 0.100 mm), (**E**) Ileal villus length (μm), (**F**) Ileal crypt depth (μm). Data are presented as mean ± SEM (*n* = 6). CON, control; MOD, model; CTC, chlortetracycline; PRO-T, probiotic treatment; PRO-P, probiotic pre-treatment; PRO, probiotic prevention plus treatment. Different lowercase letters above bars indicate significant differences among groups (*p* < 0.05).

Representative H&E-stained sections of the jejunum are shown in Figure 6A. Consistent with the histological appearance, morphometric analysis indicated shorter villi and reduced crypt depth in MOD. Jejunal villus length was 224.67 μm in MOD and 325.00 μm in CON. All intervention groups showed longer villi than MOD, including 277.50 μm in CTC, 303.33 μm in PRO-T, 314.00 μm in PRO-P, and 295.83 μm in PRO (Figure 6B). Jejunal crypt depth was 70.85 μm in MOD and 96.10 μm in CON; crypt depth remained higher than MOD in PRO-T at 80.65 μm and in PRO at 77.08 μm, with smaller increases in CTC at 71.82 μm and PRO-P at 73.02 μm (Figure 6C).

Representative ileal histology is shown in Figure 6D. Marked villus shortening was observed in MOD, which was corroborated by a villus length of 102.57 μm in MOD and 281.48 μm in CON. Interventions partially restored ileal villus length, reaching 213.63 μm in CTC, 261.38 μm in PRO-T, 254.93 μm in PRO-P, and 242.30 μm in PRO (Figure 6E). Ileal crypt depth was also reduced in MOD at 51.63 μm relative to CON at 107.93 μm. Crypt depth was higher than MOD in CTC at 72.28 μm, PRO-T at 68.60 μm, PRO-P at 72.82 μm, and PRO at 58.52 μm (Figure 6F).

### 3.7. Cecal Microbiota Composition and Diversity

**Figure 7:**
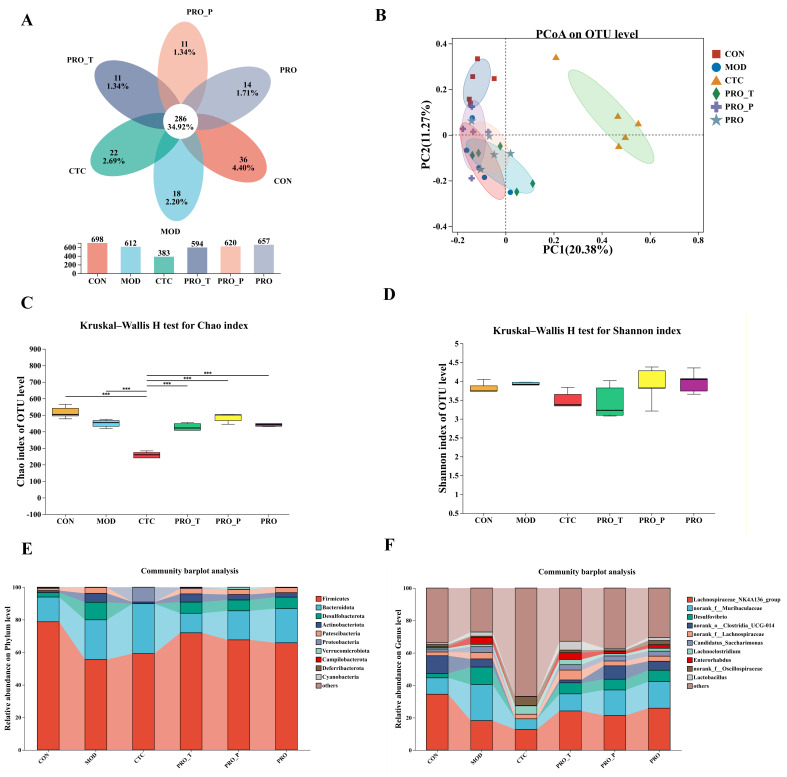
Cecal microbiota diversity and taxonomic composition across groups. (**A**) Petal plot showing shared and unique OTUs among the CON, MOD, CTC, PRO-T, PRO-P, and PRO groups; bars indicate the total number of observed OTUs in each group, (**B**) Principal coordinate analysis (PCoA) of cecal microbial communities at the OTU level, (**C**,**D**) Alpha-diversity estimated by the Chao richness index (**C**) and Shannon diversity index (**D**), (**E**) Relative abundance of dominant bacterial phyla in each group, (**F**) Relative abundance of dominant bacterial genera in each group. Statistical comparisons for alpha-diversity were performed using the Kruskal–Wallis test; significance levels are indicated as *** *p* < 0.001. Data are presented as mean ± SEM (*n* = 5). CON, control; MOD, model; CTC, chlortetracycline; PRO-T, probiotic treatment; PRO-P, probiotic pre-treatment; PRO, probiotic prevention plus treatment.

As shown in Figure 7, cecal microbiota profiles differed among the six groups (CON, MOD, CTC, PRO-T, PRO-P, and PRO). The petal plot (Figure 7A) revealed a substantial core community shared across all groups (286 OTUs, 34.92%), together with group-specific OTUs: 36 (4.40%) in CON, 18 (2.20%) in MOD, 22 (2.69%) in CTC, 11 (1.34%) in PRO-T, 11 (1.34%) in PRO-P, and 14 (1.71%) in PRO. Consistently, the total observed OTUs (bar chart in Figure 7A) ranged from 383 (CTC) to 698 (CON), with intermediate values in MOD (612), PRO-T (594), PRO-P (620), and PRO (657), indicating marked richness differences among treatments. At the OTU level, PCoA (Figure 7B) demonstrated an evident separation pattern, with CTC samples clearly displaced along PC1 (PC1 = 20.38%, PC2 = 11.27%), whereas CON and MOD clustered closer to the left side of the ordination space; notably, the probiotic-intervention groups (PRO-T/PRO-P/PRO) exhibited more dispersed distributions, suggesting greater within-group heterogeneity in community structure under different probiotic regimens. Alpha diversity further supported these differences: the Chao index (Figure 7C) showed a pronounced reduction in CTC relative to the other groups (as indicated by the multiple-comparison bars), while the probiotic-treated groups generally displayed higher richness than CTC and were closer to CON/MOD. For the Shannon index (Figure 7D), diversity tended to be lower in CTC and PRO-T, whereas PRO-P and PRO maintained comparatively higher diversity levels, approximating those of the control group.

Taxonomic profiling highlighted treatment-associated shifts at both the phylum and genus levels. At the phylum level (Figure 7E), Firmicutes remained dominant across all groups but varied widely, accounting for 78.74% in CON and dropping in MOD (55.55%) and CTC (59.20%), followed by a relative rebound in the probiotic regimens (71.99% in PRO-T, 67.63% in PRO-P, and 65.84% in PRO). In contrast, Bacteroidota increased in MOD (24.39%) and reached the highest proportion in CTC (30.64%) compared with CON (15.20%), decreased in PRO-T (12.01%), remained intermediate in PRO-P (17.85%), and was increased again in PRO (22.94%), indicating a pronounced shift in the Firmicutes-to-Bacteroidota balance across interventions. Desulfobacterota also displayed marked group dependence, being relatively low in CON (2.71%), elevated in MOD (10.82%), undetectable in CTC (0%), and maintained at moderate levels in probiotic-treated groups (6.85% in PRO-T, 6.65% in PRO-P, and 7.12% in PRO). At the genus level (Figure 7F), several taxa accounted for most of the observed compositional variation. Lachnospiraceae_NK4A136_group was abundant in CON (34.51%) but decreased in MOD (18.23%) and CTC (12.88%), and then increased under probiotic intervention (24.20% in PRO-T, 21.41% in PRO-P, and 25.90% in PRO). In parallel, norank_f__Muribaculaceae was enriched in MOD (22.24%) relative to CON (10.10%), declined in CTC (6.46%), and rose again in the probiotic groups (10.53% in PRO-T, 15.72% in PRO-P, and 16.34% in PRO). The sulfate-reducing genus *Desulfovibrio* showed a pattern consistent with Desulfobacterota, with high abundance in MOD (10.79%), absence in CTC (0%), and sustained detection in probiotic-treated mice (6.81% in PRO-T, 6.63% in PRO-P, and 7.01% in PRO; CON 2.69%). Meanwhile, norank_o__Clostridia_UCG-014 was prominent in CON (10.92%), reduced in MOD (5.01%), not detected in CTC (0%), and displayed divergent levels across probiotic strategies (1.76% in PRO-T, 8.25% in PRO-P, and 5.55% in PRO). Other genera shown in Figure 7F exhibited relatively small fluctuations and did not show pronounced group-wise differences at the resolution presented.

### 3.8. Differential Cecal Microbiota Identified by LEfSe and Kruskal–Wallis Analysis

**Figure 8:**
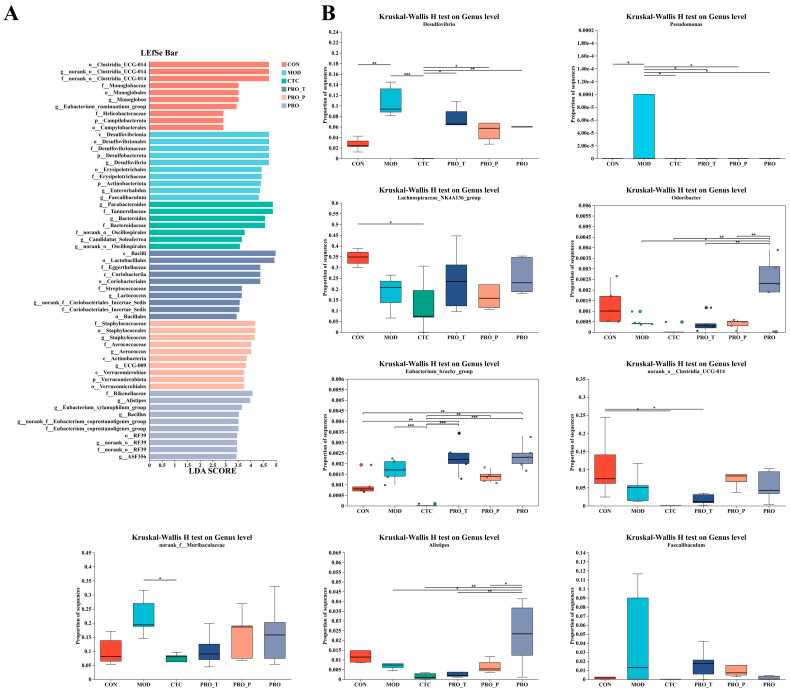
Differential taxa associated with each group identified by LEfSe and validated by genus-level comparisons. (**A**) LEfSe analysis showing group-discriminative taxa (LDA score). (**B**) Genus-level relative abundances of representative biomarkers compared among groups using the Kruskal–Wallis test. Significance levels are indicated as * *p* < 0.05, ** *p* < 0.01, and *** *p* < 0.001. Data are presented as mean ± SEM (*n* = 5). CON, control; MOD, model; CTC, chlortetracycline; PRO-T, probiotic treatment; PRO-P, probiotic pre-treatment; PRO, probiotic prevention plus treatment.

As shown in Figure 8, differentially abundant taxa across groups were identified by LEfSe and further quantified using genus-level Kruskal–Wallis analysis. In Figure 8A, LEfSe identified representative discriminative lineages for each group. The CON group was primarily characterized by features affiliated with *Clostridia_UCG-014* (multiple taxonomic ranks). The MOD group showed enrichment of sulfate-reduction–associated lineages, prominently including g__*Desulfovibrio* and higher-rank Desulfobacterota/Desulfovibrionia-related features. In contrast, the CTC group was mainly associated with Bacteroidota-related lineages such as g__*Bacteroides* and allied taxa. Probiotic regimens also showed group-specific signatures, with PRO-T linked to Bacilli/Lactobacillales-related features, and PRO exhibiting enrichment of g__*Alistipes* and several additional discriminative lineages.

In Figure 8B, genus-level testing further substantiated these group differences. *Desulfovibrio* differed significantly among groups, being markedly elevated in MOD compared with CON (*p* < 0.01) and showing near-complete depletion in CTC (*p* < 0.001); probiotic-treated groups (PRO-T, PRO-P and PRO) displayed intermediate levels relative to MOD (*p* < 0.05). *Pseudomonas* was detected predominantly in MOD, with significantly higher abundance than CON, CTC, and probiotic groups (*p* < 0.05). Lachnospiraceae_NK4A136_group was highest in CON and reduced in MOD and CTC, with probiotic regimens showing a partial rebound toward higher levels (overall group effect *p* < 0.05). In addition, *Odoribacter* showed a pronounced increase in PRO compared with other groups (*p* < 0.01), whereas the remaining groups largely remained low. For the other genera shown, including the *Eubacterium* (brachy group), norank_o__Clostridia_UCG-014, norank_f__Muribaculaceae, *Alistipes*, and *Faecalibaculum*, their distributions also varied across groups, with several taxa displaying group-dependent shifts and/or broader inter-individual dispersion under probiotic regimens.

### 3.9. Spearman Correlation Analysis

**Figure 9:**
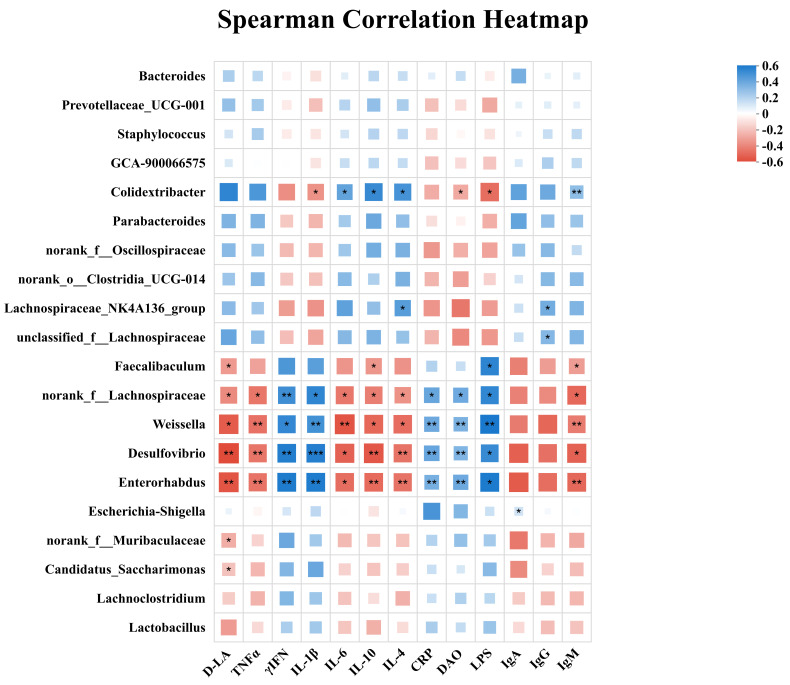
Spearman’s rank correlation heatmap showing relationships between key bacterial taxa and serum indices. Correlation coefficients are indicated by the color scale (blue, positive; red, negative), with stronger colors reflecting larger absolute correlations. Significance levels are indicated as * *p* < 0.05, ** *p* < 0.01, and *** *p* < 0.001. *p* values were corrected for multiple testing using the Benjamini–Hochberg false discovery rate (FDR) procedure. *n* = 5 per group.

Spearman correlation analysis revealed significant associations between key intestinal bacterial genera and host inflammatory, barrier, and immune-related indices (Figure 9). Several taxa exhibited strong correlations with pro-inflammatory cytokines and endotoxin-related markers. Notably, *Desulfovibrio* showed significant positive correlations with TNF-α, IFN-γ, IL-1β, IL-6, CRP, and LPS (*p* < 0.05 or *p* < 0.01), while being negatively correlated with immunoglobulin levels, particularly IgA and IgG (*p* < 0.05). Similarly, *Weissella* and *Enterorhabdus* were positively associated with multiple inflammatory markers, including TNF-α, IL-1β, IL-6, DAO, and LPS, and displayed significant negative correlations with IgA and IgG (*p* < 0.01), indicating a close association with intestinal inflammation and barrier impairment.

In contrast, several putative beneficial or commensal taxa were negatively correlated with inflammatory indicators. Lachnospiraceae-related genera, including Lachnospiraceae_NK4A136_group, norank_f__Lachnospiraceae, and unclassified_f__Lachnospiraceae, showed significant negative correlations with pro-inflammatory cytokines (TNF-α, IL-1β, IL-6) and LPS, while being positively correlated with IgA, IgG, and IgM (*p* < 0.05 or *p* < 0.01). In addition, *Faecalibaculum* exhibited negative associations with TNF-α, IL-6, and LPS, and positive correlations with IgA and IgM (*p* < 0.05), whereas *Colidextribacter* showed a mixed pattern, being negatively correlated with IL-1β and IL-6 but positively correlated with immunoglobulin levels (*p* < 0.01). Collectively, these correlations highlight distinct relationships between intestinal microbial taxa and host inflammatory status, mucosal barrier function, and systemic immune responses.

## 4. Discussion

This study demonstrates that feline-derived *L. agilis* ZY25 and *L. salivarius* ZY35 exhibit protective effects against EPEC infection in mice, as evidenced by the alleviation of diarrhea, mitigation of weight loss, restoration of intestinal barrier function, modulation of inflammatory cytokines, and normalization of gut microbiota composition. These findings highlight the potential of feline-origin probiotics as alternative or complementary interventions to antibiotics for controlling enteric bacterial infections. Previous research has reported that *L. salivarius* possesses potent antibacterial and anti-inflammatory activities, capable of suppressing intestinal inflammation and maintaining epithelial integrity through multiple mechanisms, including enhancement of tight junction proteins and modulation of host immune signaling [24]. Moreover, *Ligilactobacillus* species have shown the ability to inhibit pathogen colonization by producing organic acids, bacteriocins, and biosurfactants, which reduce adhesion of pathogenic *E. coli* to intestinal epithelial cells [25]. These effects align with the current findings, where preventive and therapeutic probiotic administration alleviated EPEC-induced pathophysiological changes with efficacy comparable to antibiotic treatment in several evaluated parameters. From a broader perspective, the study contributes to growing evidence that host-specific *Ligilactobacillus* strains, especially those isolated from carnivores, may exhibit enhanced ecological fitness and colonization capacity in mammalian intestines [26]. Notably, feline-derived *L. salivarius* and *L. agilis* may provide unique probiotic traits derived from adaptation to carnivore-associated microbiota, including strong tolerance to bile salts and efficient carbohydrate metabolism [27]. This host-origin adaptation may underlie their superior efficacy in maintaining gut homeostasis and reducing pathogen-induced inflammation observed in the present study. Together, these findings underscore the translational potential of feline-derived *Ligilactobacillus* strains as promising next-generation probiotics for preventing and mitigating enteric infections.

EPEC infection typically induces diarrhea and growth suppression by damaging intestinal epithelia and disrupting nutrient absorption. In this study, supplementation with feline-derived *L. agilis* ZY25 and *L. salivarius* ZY35 effectively alleviated EPEC-induced diarrhea and weight loss, reflecting their robust protective potential against enteric infection. Our previous findings confirmed that both strains inhibit EPEC primarily through organic acid production, particularly lactic and acetic acids, which lower gut luminal pH and suppress pathogen proliferation. Beyond this established acid-mediated mechanism, the current in vivo results suggest that multiple complementary actions jointly contribute to the observed physiological protection. Notably, genomic analyses have revealed that *L. salivarius* encodes diverse bacteriocin clusters, such as *salivaricin* and *Abp118*, capable of disrupting bacterial membranes and interfering with toxin secretion, thereby mitigating epithelial injury and fluid secretion [28,29]. Moreover, both *L. agilis* and *L. salivarius* exhibit strong adhesive and biofilm-forming capacities that facilitate colonization and competitive exclusion of pathogens, effectively maintaining mucosal stability and preventing microbial displacement [30]. In addition to these direct antimicrobial and barrier-protective effects, recent evidence indicates that *L. salivarius* metabolites can attenuate intestinal inflammation by downregulating the TLR4/NF-κB pathway and modulating cytokine expression, thereby limiting epithelial permeability and systemic immune activation [31,32]. Collectively, these synergistic actions—acid-driven pathogen suppression, bacteriocin-mediated antagonism, epithelial colonization, and immune modulation—provide a comprehensive explanation for the probiotic-mediated mitigation of diarrhea and maintenance of body weight in EPEC-challenged mice.

Administration of *L. agilis* ZY25 and *L. salivarius* ZY35 effectively alleviated EPEC-induced intestinal dysfunction, as reflected by reduced serum D-lactate, DAO, and LPS levels, together with improved intestinal histomorphology and attenuation of inflammatory responses. These findings support a protective effect on barrier-related injury and endotoxin translocation in EPEC-challenged mice. However, the present study did not directly assess tight junction proteins (e.g., ZO-1, occludin, or claudin-1), mucin-related markers such as MUC2, or other molecular indices of epithelial integrity. Rather than demonstrating direct restoration of tight junction architecture, the current data indicate that probiotic supplementation was associated with improved permeability-related serum biomarkers and histological recovery. Previous studies have reported that *L. salivarius* strains can modulate epithelial tight junctions and inflammatory signaling, but such mechanisms were not directly verified in the present work [33,34,35,36]. Future studies should incorporate direct epithelial and mucosal markers, including ZO-1, occludin, claudin-1, and MUC2, to clarify the molecular basis of the barrier-related protection observed here.

Beyond local intestinal protection, *L. agilis* and *L. salivarius* supplementation also influenced systemic immune responses, as reflected by increased IL-4, IL-10, and immunoglobulin levels and decreased TNF-α, IL-6, IFN-γ, and CRP. From an immunological perspective, this pattern is consistent with partial correction of an EPEC-induced pro-inflammatory shift toward a more Th1-dominant milieu. In intestinal inflammation, TNF-α and IFN-γ are closely associated with Th1-driven immune activation and can aggravate epithelial injury, macrophage activation, and barrier dysfunction, whereas IL-4 and IL-10 are more closely linked to Th2/regulatory responses that help counterbalance excessive Th1-associated inflammation. IL-6 further amplifies inflammatory cascades and contributes to sustained mucosal immune activation [37,38,39]. Accordingly, the cytokine profile observed in the MOD group suggests a disturbed inflammatory equilibrium, while probiotic administration—particularly in the continuous supplementation group—shifted this profile toward a more regulated immune state rather than simply suppressing inflammation non-specifically.

This interpretation is also relevant in the context of host-adapted probiotics for companion animals. Recent feline research has emphasized that many probiotic candidates used in cats are not cat-derived and may therefore show reduced host adaptation, whereas feline-origin strains are increasingly considered more relevant for target-species gastrointestinal applications [40]. Consistent with this concept, a randomized placebo-controlled trial demonstrated that a feline-origin *Enterococcus hirae* probiotic reduced diarrhea risk in shelter kittens, supporting the translational value of host-adapted probiotic strategies in cats [19]. In addition, recent feline studies and companion-animal reviews continue to highlight the growing interest in probiotic applications in cats and dogs [18,20,41]. Collectively, these data strengthen the interpretation that ZY25 and ZY35 may alleviate EPEC-associated intestinal inflammation not only through antimicrobial and microbiota-mediated effects, but also through modulation of cytokine networks linked to Th1/Th2 immune balance. Future studies in cats should further verify whether these feline-derived strains produce comparable immunoregulatory effects under target-species conditions.

The 16S rRNA sequencing results revealed that *L. agilis* ZY25 and *L. salivarius* ZY35 supplementation reshaped the intestinal microbial community in EPEC-infected mice, restoring microbial diversity and stabilizing the Firmicutes/Bacteroidota ratio, which is a key indicator of intestinal ecological balance. EPEC challenge significantly decreased beneficial taxa such as Lachnospiraceae and Ruminococcaceae while increasing the relative abundance of *Desulfovibrio* and other opportunistic pathogens associated with lipopolysaccharide (LPS) production and mucosal inflammation. These dysbiotic features have been commonly observed in infection and colitis models, and their normalization by probiotic intervention indicates partial restoration of intestinal homeostasis [42,43]. The enrichment of Lachnospiraceae_NK4A136_group and Muribaculaceae in the probiotic-treated groups suggests enhanced short-chain fatty acid (SCFA) production, particularly butyrate, which is known to support epithelial energy metabolism and reinforce tight junction integrity [44].

Additionally, probiotics likely suppressed *Desulfovibrio*, a sulfate-reducing bacterium linked to hydrogen sulfide (H_2_S) overproduction and mucosal barrier disruption. Decreased abundance of Desulfovibrionaceae has been associated with lower systemic endotoxin load and improved epithelial repair capacity [45]. Restoration of SCFA-producing commensals also plays a pivotal role in regulating host immune signaling by activating G-protein-coupled receptors (GPR41/43) and inhibiting histone deacetylases, which collectively suppress NF-κB-mediated inflammation and promote intestinal tolerance [46]. The positive correlation between the abundance of Lachnospiraceae and improved barrier markers observed here aligns with reports that SCFA-producing bacteria modulate epithelial gene expression and enhance mucosal thickness [47].

Moreover, the partial recovery of microbial community composition after probiotic administration indicates that *L. agilis* and *L. salivarius* act not only as transient colonizers but also as ecological modulators capable of promoting beneficial bacterial cross-feeding. This microbial reconfiguration likely contributes to the observed reduction in inflammatory cytokines and serum LPS. Previous work has shown that restoration of *Firmicutes* taxa and reduction in *Proteobacteria* abundance are associated with improved intestinal integrity and lower oxidative stress in infection and inflammatory models [48]. Collectively, these findings suggest that *L. agilis* and *L. salivarius* mitigate EPEC-induced dysbiosis by restoring beneficial commensal populations, suppressing inflammatory pathobionts, and enhancing SCFA-mediated metabolic signaling, thereby supporting intestinal barrier and immune homeostasis. It should also be noted that the present microbiota analysis was based on OTU clustering rather than an ASV-based denoising pipeline; therefore, the current findings should be interpreted as community-level evidence, and future ASV-based reanalysis may further improve taxonomic resolution.

A major strength of this study lies in its systematic comparison of three probiotic intervention strategies—preventive (PRO-P), therapeutic (PRO-T), and probiotic prevention plus treatment (PRO)—which collectively reveal how the timing and continuity of *L. agilis* ZY25 and *L. salivarius* ZY35 administration influence their protective efficacy against EPEC-induced intestinal injury. Among these, all probiotic regimens exerted beneficial effects to varying degrees; however, the combined strategy (PRO) consistently produced the most pronounced improvements across multiple physiological and immunological parameters, including body weight recovery, diarrhea attenuation, serum barrier markers (D-LA, DAO, LPS), and cytokine balance. The preventive regimen (PRO-P) moderately alleviated diarrhea and maintained epithelial integrity, suggesting that early exposure to probiotics may enhance mucosal readiness and colonization resistance. Meanwhile, the therapeutic regimen (PRO-T) facilitated recovery after infection, likely by restoring microbial homeostasis, reducing endotoxemia, and modulating inflammatory signaling. The superior overall performance of the PRO group indicates that the regimen of continuous probiotic administration before and after infection provides cumulative protection effects, likely attributable to prolonged host exposure and sustained modulation of the intestinal environment, rather than to transient passage alone. These observations align with previous findings that prolonged *L. salivarius* administration confers more stable anti-inflammatory and microbiota-balancing effects than single-phase interventions [29,49,50]. Collectively, these results underscore that the timing and duration of probiotic exposure are critical determinants of their clinical efficacy and highlight the potential advantages of sustained supplementation regimens for managing intestinal infection and maintaining gut homeostasis.

Although the present study provides in vivo evidence that feline-derived *L. agilis* ZY25 and *L. salivarius* ZY35 can alleviate EPEC-induced intestinal injury in C57BL/6 mice, caution is required when translating these findings to cats. In this study, the murine EPEC challenge model was used as a controlled proof-of-concept platform for evaluating anti-infective and gut-protective effects under standardized experimental conditions. However, this model does not fully reproduce the intestinal ecosystem, dietary physiology, immune environment, or microbial community structure of the feline host. In particular, cats are obligate carnivores, and their gut microbiota is strongly influenced by age, diet composition, and gastrointestinal disease status, whereas the gut microbiota of laboratory mice is shaped by rodent-specific ecology, standardized chow, coprophagy, housing conditions, and cage effects. These interspecies differences may influence probiotic colonization dynamics, ecological fitness, and the magnitude or mechanism of host responses [51]. Experimental studies have further shown that native gut bacteria may exhibit a competitive “home-site advantage” in their original host background, indicating that microbial adaptation and host responsiveness are partly species dependent. Therefore, the present findings should be interpreted as proof-of-concept evidence of biological activity in a controlled mammalian system, rather than as definitive evidence of clinical efficacy in cats. Notably, feline-origin probiotic administration has already shown preventive benefits in kittens under field-relevant conditions, further underscoring the importance of validation in the target species itself [52].

Furthermore, while the continuous supplementation regimen (PRO) showed the greatest efficacy among the three intervention strategies tested in this study, several methodological limitations should be acknowledged. Our experimental design was not intended to establish a dose–response relationship, nor did it include strain-specific quantification (e.g., qPCR) to confirm the active colonization or persistence of *L. agilis* ZY25 and *L. salivarius* ZY35 in the murine gut. Therefore, the superior protective effects observed in the PRO group should be attributed to the continuous administration regimen itself, namely the longer and uninterrupted exposure of the host to the probiotics, rather than to proven long-term colonization or mucosal association. The precise mechanisms by which sustained intake confers cumulative benefits, whether through transient metabolic effects, immune modulation, or ecological interactions, remain to be elucidated. Future studies should combine molecular tracking approaches with metagenomic and metabolomic analyses to verify strain colonization dynamics, optimize dosing regimens, and assess host responses and microbiota remodeling in feline models or naturally occurring feline gastrointestinal disorders.

## 5. Conclusions

In conclusion, the present study demonstrates that feline-derived *L. agilis* ZY25 and *L. salivarius* ZY35 confer significant protection against EPEC-induced intestinal injury in mice. EPEC challenge resulted in growth suppression, diarrhea, epithelial damage, systemic inflammation, and microbial dysbiosis, whereas probiotic supplementation effectively alleviated these pathological changes. Improvements were evidenced by restored villus morphology, reduced serum D-lactate, DAO, and LPS levels, rebalanced pro- and anti-inflammatory cytokines, enhanced immunoglobulin production, and partial recovery of gut microbial diversity with enrichment of beneficial taxa and suppression of inflammation-associated genera. Notably, the continuous supplementation regimen before and after infection produced the most consistent protective effects, underscoring the importance of sustained probiotic administration for maximizing protective efficacy. Collectively, these findings indicate that ZY25 and ZY35 alleviate EPEC-induced intestinal injury in mice and are associated with improved barrier-related biomarkers, immune balance, and microbial homeostasis. Among the three intervention strategies tested, continuous supplementation showed the most consistent protective effects. However, direct molecular evidence for tight junction or mucin regulation was not obtained in the present study, and the underlying epithelial mechanisms therefore remain to be further clarified. In addition, because this work was conducted in a murine challenge model, further validation in cats is required before efficacy in feline gastrointestinal health management can be concluded.

## Figures and Tables

**Table 1:** Three-phase experimental design and intervention schedule for the murine EPEC challenge study.

Group	Days 1–7	Days 8–14	Days 15–21
CON	PBS	PBS	PBS
MOD	PBS	PBS + 1×10^9^ CFU EPEC	PBS
CTC	PBS	PBS + 1×10^9^ CFU EPEC	PBS + 2 mg Chlorotetracycline
PRO-T	PBS	PBS + 1×10^9^ CFU EPEC	PBS + 1 × 10^9^ CFU probiotics
PRO-P	PBS + 1 × 10^9^ CFU probiotics	PBS + 1 × 10^9^ CFU EPEC + 1 × 10^9^ CFU probiotics	PBS
PRO	PBS + 1 × 10^9^ CFU probiotics	PBS + 1 × 10^9^ CFU EPEC + 1 × 10^9^ CFU probiotics	PBS + 1 × 10^9^ CFU probiotics

## Data Availability

The 16S rRNA sequencing data have been deposited in the NCBI Sequence Read Archive (SRA) under accession number PRJNA 1422971. Other datasets generated during this study are available from the corresponding author upon reasonable request.

## Supplementary Materials

The following supporting information can be downloaded at: https://www.mdpi.com/article/10.3390/microorganisms14030679/s1, Table S1: Body weight changes (g) during the experimental period; Table S2: Time course of the fecal scoring index reflecting diarrhea severity.

## Abbreviations

EPEC: *enteropathogenic Escherichia coli*, DAO: diamine oxidase, D-LA: D-lactate, LPS: lipopolysaccharide, CRP: C-reactive protein, IgA: immunoglobulin A, IgG: immunoglobulin G, IgM: immunoglobulin M, TNF-α: tumor necrosis factor-α, IL-1β: interleukin-1β, IL-4: interleukin-4, IL-6: interleukin-6, IL-10: interleukin-10, IFN-γ: interferon-γ, IACUC: Institutional Animal Care and Use Committee, PBS: phosphate-buffered saline, CFU: colony-forming unit, CTC: chlortetracycline, CON: normal control, MOD: EPEC-infected model, PRO-T: probiotic treatment, PRO-P: probiotic pre-treatment, PRO: probiotic prevention plus treatment, H&E: hematoxylin and eosin, NIH: National Institutes of Health, ELISA: enzyme-linked immunosorbent assay, 16S rRNA: 16S ribosomal RNA, DNA: deoxyribonucleic acid, PCR: polymerase chain reaction, OTU: operational taxonomic unit, OTUs: operational taxonomic units, PCoA: principal coordinate analysis, PC1: principal coordinate 1, PC2: principal coordinate 2, PERMANOVA: permutational multivariate analysis of variance, ANOVA: analysis of variance, SEM: standard error of the mean, SPSS: Statistical Package for the Social Sciences, LEfSe: linear discriminant analysis effect size, LDA: linear discriminant analysis, FDR: false discovery rate, SCFA: short-chain fatty acid.
