# Supplementary material for: Feline-Derived Ligilactobacillus agilis ZY25 and Ligilactobacillus salivarius ZY35 Alleviate Enteropathogenic Escherichia coli-Induced Intestinal Injury and Microbial Dysbiosis in Mice
Source: Microorganisms. 2026 Mar 17;14(3):679. doi: 10.3390/microorganisms14030679 (PMC13028766; doi:10.3390/microorganisms14030679)
Supplement: Supplementary file 1 [file microorganisms-14-00679-s001.zip › microorganisms-4178917-supplementary.pdf]

Supplementary Materials

Table S1. Body weight changes (g) during the experimental period.

Days	Group					
	CON	MOD	CTC	PRO-T	PRO-P	PRO
1	23.14±0.43^{a}	23.40±0.25^{a}	23.26±0.36^{a}	23.30±0.19^{a}	23.36±0.18^{a}	23.12±0.32^{a}
3	23.84±0.28^{a}	23.24±0.43^{a}	23.66±0.42^{a}	23.01±0.18^{a}	23.54±0.37^{a}	23.60±0.35^{a}
5	23.92±0.14^{a}	23.48±0.19^{a}	23.04±0.13^{a}	23.21±0.31^{a}	23.88±0.39^{a}	23.98±0.25^{a}
7	24.18±0.33^{a}	23.38±0.17^{b}	23.18±0.02^{b}	23.42±0.11^{b}	23.62±0.42^{b}	24.06±0.22^{a}
9	24.88±0.25^{a}	22.62±0.23^{c}	23.22±0.38^{b}	22.92±0.19^{b}	22.86±0.26^{b}	24.22±0.37^{a}
11	25.36±0.20^{a}	22.36±0.19^{c}	23.12±0.19^{b}	22.31±0.20^{c}	22.50±0.31^{c}	23.42±0.31^{b}
13	25.52±0.27^{a}	21.76±0.43^{c}	22.86±0.33^{b}	22.01±0.19^{c}	22.22±0.31^{c}	22.76±0.34^{b}
15	26.16±0.29^{a}	22.00±0.44^{b}	22.88±0.31^{b}	22.21±0.27^{b}	22.84±0.31^{b}	22.96±0.38^{b}
17	26.66±0.28^{a}	22.38±0.37^{b}	23.16±0.35^{b}	22.72±0.19^{b}	22.94±0.37^{b}	23.28±0.21^{b}
19	26.83±0.39^{a}	22.66±0.36^{c}	23.56±0.28^{b}	22.90±0.30^{b}	23.24±0.25^{b}	23.52±0.37^{b}
21	27.40±0.44^{a}	22.90±0.29^{c}	23.92±0.42^{b}	23.34±0.15^{c}	23.49±0.22^{c}	24.18±0.39^{b}

Note: Data are presented as mean ± SEM. Different superscript letters within the same row indicate significant differences among groups at the same time point ($\boldsymbol{p} < 0.05$), while the same superscript letter indicates no significant difference ($p \geq 0.05$). CON, control; MOD, model; CTC, chlortetracycline; PRO-T, probiotic treatment; PRO-P, probiotic pre-treatment; PRO, probiotic prevention plus treatment.

Table S2. Time course of the fecal scoring index reflecting diarrhea severity.

Days	Group					
	CON	MOD	CTC	PRO-T	PRO-P	PRO
1	ND	ND	ND	ND	ND	ND
3	ND	ND	ND	ND	ND	ND
5	ND	ND	ND	ND	ND	ND
7	ND	ND	ND	ND	ND	ND
9	ND	2.10 ± 0.06^{a}	1.20 ± 0.14^{b}	1.90 ± 0.05^{a}	1.80 ± 0.26^{a}	1.40 ± 0.13^{b}
11	ND	2.40 ± 0.12^{a}	1.00 ± 0.04^{b}	2.00 ± 0.03^{a}	1.80 ± 0.14^{a}	1.20 ± 0.09^{b}
13	ND	1.90 ± 0.09^{a}	0.60 ± 0.12^{b}	1.40 ± 0.14^{a}	1.20 ± 0.09^{a}	0.80 ± 0.06^{b}
15	ND	0.90 ± 0.03^{a}	0.50 ± 0.16^{b}	0.60 ± 0.08^{b}	0.40 ± 0.05^{b}	0.40 ± 0.02^{b}
17	ND	0.60 ± 0.04	ND	ND	ND	ND
19	ND	ND	ND	ND	ND	ND
21	ND	ND	ND	ND	ND	ND

Note: Data are presented as mean ± SEM. Different superscript letters within the same row indicate significant differences among groups at the same time point ($p < 0.05$), while the same superscript letter indicates no significant difference ($p \geq 0.05$). ND indicates that no diarrhea was detected. CON, control; MOD, model; CTC, chlortetracycline; PRO-T, probiotic treatment; PRO-P, probiotic pre-treatment; PRO, probiotic prevention plus treatment.
